# Tracking Cholesterol/Sphingomyelin-Rich Membrane Domains with the Ostreolysin A-mCherry Protein

**DOI:** 10.1371/journal.pone.0092783

**Published:** 2014-03-24

**Authors:** Matej Skočaj, Nataša Resnik, Maja Grundner, Katja Ota, Nejc Rojko, Vesna Hodnik, Gregor Anderluh, Andrzej Sobota, Peter Maček, Peter Veranič, Kristina Sepčić

**Affiliations:** 1 Department of Biology, Biotechnical Faculty, University of Ljubljana, Ljubljana, Slovenia; 2 Institute of Cell Biology, Faculty of Medicine, University of Ljubljana, Ljubljana, Slovenia; 3 Institute of Biophysics, Faculty of Medicine, University of Ljubljana, Ljubljana, Slovenia; 4 National Institute of Chemistry, Ljubljana, Slovenia; 5 Nencki Institute of Experimental Biology, Warsaw, Poland; Institut Curie, France

## Abstract

Ostreolysin A (OlyA) is an ∼15-kDa protein that has been shown to bind selectively to membranes rich in cholesterol and sphingomyelin. In this study, we investigated whether OlyA fluorescently tagged at the C-terminal with mCherry (OlyA-mCherry) labels cholesterol/sphingomyelin domains in artificial membrane systems and in membranes of Madin-Darby canine kidney (MDCK) epithelial cells. OlyA-mCherry showed similar lipid binding characteristics to non-tagged OlyA. OlyA-mCherry also stained cholesterol/sphingomyelin domains in the plasma membranes of both fixed and living MDCK cells, and in the living cells, this staining was abolished by pretreatment with either methyl-β-cyclodextrin or sphingomyelinase. Double labelling of MDCK cells with OlyA-mCherry and the sphingomyelin-specific markers equinatoxin II–Alexa488 and GST-lysenin, the cholera toxin B subunit as a probe that binds to the ganglioside G_M1_, or the cholesterol-specific D4 domain of perfringolysin O fused with EGFP, showed different patterns of binding and distribution of OlyA-mCherry in comparison with these other proteins. Furthermore, we show that OlyA-mCherry is internalised in living MDCK cells, and within 90 min it reaches the juxtanuclear region *via* caveolin-1–positive structures. No binding to membranes could be seen when OlyA-mCherry was expressed in MDCK cells. Altogether, these data clearly indicate that OlyA-mCherry is a promising tool for labelling a distinct pool of cholesterol/sphingomyelin membrane domains in living and fixed cells, and for following these domains when they are apparently internalised by the cell.

## Introduction

Biological membranes are composed of thousands of species of proteins and lipids [1]. While for the proteins, the diverse sets of functions are largely known, the roles of the several thousand different species of lipids are still not exactly clear. Lipids in biological membranes were first considered as a homogenous mixture, but later, in the 1990's, the concept of membrane rafts was introduced [2]. Membrane rafts are currently defined as dynamic, nanoscale-sized, sterol- and sphingolipid-enriched assemblies. They can coalesce into larger, more stable, raft domains through specific lipid–lipid, protein–lipid and protein–protein interactions [1]. Clustering of membrane rafts enhances the inclusion of proteins that can specifically partition into rafts, while it excludes those that are segregated away [3]. Similarly, in this model, cholesterol and sphingomyelin (SM) have pivotal roles for the separation of the membrane lipid domains into co-existing liquid-disordered (*l_d_*) and liquid-ordered (*l_0_*) domains, where *l_0_* domains correspond to the raft phase [4]. In contrast to lipids in *l_d_* domains, those in the *l_0_* phase are more resistant to solubilisation by detergents [5].

Experimental evidence over the past few years has shown that rafts are involved in numerous biological functions, such as exocytosis, endocytosis, cell signalling, pathogen entry, and attachment of various molecular ligands [1], [2], [6]–[9]. They have also been shown to participate in the transduction of various signals that are important in a variety of disease conditions; e.g., Alzheimer's disease, Parkinson's disease, cardiovascular and prion diseases, systemic lupus erythematosus, and acquired immunodeficiency syndrome [10]. Therefore, the development of new approaches, techniques and tools that allow ‘visualisation’ of these membrane domains is of great importance.

Membrane rafts are difficult to visualise due to their temporal instability and small size [11]. Several modern scanning and optical microscopy approaches have been used recently to visualise these membrane domains [12], [13]. Also, new fluorescently labelled probes have been developed to obtain more insight into particular membrane lipids and/or lipid domains, such as lipid analogues, lipid-binding proteins, and antibodies [14], or non-toxic recombinant derivatives of natural toxins. Some protein toxins are candidates for raft markers, as they can interact with specific molecules that are enriched in these membrane domains; e.g., cholesterol, SM, ceramides, gangliosides, or the glycan core of glycophosphatidylinositol-anchored proteins [15]–[17]. Among the non-toxic fluorescently labelled toxin derivatives, the cholera toxin B subunit (CT-B) that binds to the ganglioside G_M1_ that is enriched in rafts has long been the probe of choice for labelling membrane rafts [18]. As one of the major lipids of the vertebrate plasma membrane, SM is mainly located in the plasmalemma outer leaflet, and it can be specifically recognised by lysenin [19], [20], a protein that is secreted through the dorsal pores of the earthworm *Eisenia foetida* [21], and by equinatoxin II (EqTII), a cytolysin from the sea anemone *Actinia equina* [22]. Although both of these toxins have been shown to bind to SM, they interact with distinct membrane pools of this lipid [23]. The fluorescently-labelled D4 domain of perfringolysin O (PFO), a cytolysin from the Gram-positive bacterium *Clostridium perfringens*, was designed for selective labelling of cholesterol-enriched membrane domains [24], [25]. In contrast to these proteins that bind selectively to either cholesterol or SM, it was shown that the 15-kDa aegerolysins [26], ostreolysin A (OlyA) and a pleurotolysin A orthologue, from the mushrooms *Pleurotus ostreatus* and *P. eryngii*, respectively, bind selectively to membranes only if these are enriched in both cholesterol and SM [27], [28]. These two highly similar proteins are not cytolytic themselves, although they are a component of a cytolytic binary pore complex, as was previously reported for *P. ostreatus* pleurotolysin A [29], and *P. eryngii* erylysin A [30]. Specifically, binding of OlyA to cholesterol/SM is essential for recruitment of the membrane attack complex/perforin-domain-containing 59-kDa protein pleurotolysin B (PlyB) onto cholesterol/SM-rich model lipid membranes and onto cell membranes to form the binary pore-complex that is permeable to solutes [27], [31]. This specific recognition of cholesterol/SM-enriched membrane domains means that the non-cytolytic OlyA and similar mushroom proteins are potential tools for the detection of cellular raft-like membrane domains. Indeed, pleurotolysin A2 (PlyA2), from the mushroom *P. eryngii*, which has 80% identity with OlyA, was fused very recently with enhanced green fluorescent protein (PlyA2-EGFP) and shown to selectively label cholesterol/SM-rich domains in membranes of HeLa cells [28].

Here, we describe the preparation and lipid-binding characteristics of OlyA fused with the fluorescent mCherry protein (OlyA-mCherry). We used Madin-Darby canine kidney (MDCK) epithelial cells to examine the labelling potential of ectopically applied OlyA-mCherry for cholesterol/SM domains in the external leaflet of the plasma membrane, and intracellularly expressed OlyA-mCherry for cholesterol/SM domains in cytosol-facing membranes. We show that OlyA-mCherry is not toxic to the cells, and it labels specific cholesterol/SM-rich plasma membrane domains that are not detected using other lipid-specific protein probes, in both fixed and living cells. Moreover, we have addressed question whether the preference of OlyA for high cholesterol/SM domains in the plasmalemma might promote OlyA endocytosis. To this end, we explored the cell internalisation of OlyA-mCherry *via* raft-dependent, i.e., caveolin-1–dependent and flotillin-1–dependent, endocytotic pathways [32], [33], and/or the clathrin-dependent pathway, which is raft-independent [34]. Our data indicate that OlyA can also be used to direct a fused protein of choice as a cargo *via* caveolae into the cell endosomal recycling system.

## Experimental Procedures

### 1. Reagents and materials

#### 1.1. Plasmids and restriction enzymes

The restriction enzyme *SmaI* was from New England Biolabs (USA), and the FastDigest restriction enzymes *Bam*HI, *Bgl*II, *Mlu*I, *Nde*I, *Xho*I, rapid DNA ligation kits, GeneJET PCR purification kits, GeneJET gel extraction kits, GeneJET plasmid miniprep kits, and PageRuler prestained protein ladder were all from Fermentas (Thermo Scientific, USA). The pET plasmids were from Novagen (Merck, USA), and the pmCherry plasmids from Clontech (USA). Oligonucleotide primers for the OlyA (NCBI acc. code: AGH25589) constructs were synthesised by Sigma-Aldrich (USA), while the gene coding the D4 domain of PFO was synthesised by GenScript (USA).

#### 1.2. Lipids

Wool grease cholesterol, porcine brain SM, and 1-palmitoyl-2-oleoyl-*sn*-glycero-3-phosphocholine (POPC) were from Avanti Polar Lipids (Alabaster, USA). Total lipids from bovine erythrocyte membranes were extracted according to Bligh and Dyer [35], and stored under liquid nitrogen at −80°C.

#### 1.3. Antibodies and proteins

**Figure 1:**
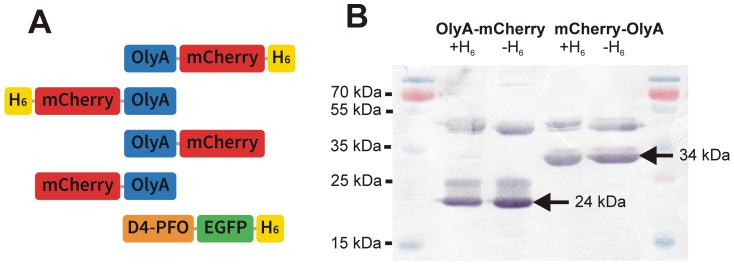
OlyA and perfringolysin O variants investigated in this study. (**A**) Domain structures and tags of the fluorescently fused OlyA and PFO. (**B**) Western blotting of recombinant OlyA variants expressed in bacteria. H_6_, hexa-histidine; EGFP, enhanced green fluorescent protein; other abbreviations as used in the main text. Calculated relative molecular masses: *M*r: OlyA-mCherry-H_6_, 44,318.58 Da; H_6_-mCherry-OlyA, 43,409.66 Da; OlyA-mCherry, 42,489.69 Da; mCherry-OlyA, 42,035.15 Da. Detection of the proteins in Western blotting was performed using polyclonal anti-OlyA antibodies. Arrows denote spontaneously cleaved mCherry-tagged OlyA products [46].

The mouse anti–caveolin-1 and anti–cathepsin-L antibodies were from Abcam (UK), the rabbit anti-flotillin, mouse anti-phosphotyrosine, and rabbit anti-glutathione-S-transferase (GST) antibodies were from Sigma-Aldrich (USA), the mouse anti-clathrin (heavy chain), anti-EEA1, and anti-GM130 antibodies were from BD Biosciences (USA), the mouse anti-P230 antibody was from Dako (Denmark), the mouse anti-giantin antibody was from Alexis Biochemicals (Enzo Life Sciences, USA), and the rabbit anti-occludin antibody was from Zymed (USA). The rabbit polyclonal and monoclonal anti-OlyA antibodies were produced as described by Berne et al. [36] and Ota et al. [27], respectively. The secondary Alexa Fluor 488-conjugated goat anti-rabbit or anti-mouse antibodies were from Molecular Probes (Life Technologies, USA), and the HRP-conjugated anti-rabbit and anti-mouse antibodies were from Santa Cruz Biotechnology (USA). Sphingomyelinase was from Sigma-Aldrich (USA). Glutathione-S-transferase-lysenin (GST-lysenin) was prepared as indicated [37]. For the use in co-localisation studies, a fluorescently labelled non-lytic triple Cys EqTII mutant (EqTII-Alexa488) was prepared as previously described [38]. The fluorescently labelled D4 domain of PFO (NCBI acc. number CP000246), D4-PFO-EGFP, was constructed as reported by Shimada et al. [25] (see **File S1**). When labelling the cells with cholera toxin subunit B, CT-B-Alexa488, Vybrant Lipid Raft Labelling kits were used (Molecular Probes, Life Technologies, USA). Native OlyA and the recombinant N-terminally truncated mature form of PlyB (Δ48PlyB) were prepared as described by [27]. The fluorescently labelled OlyA variants were constructed for expression in bacteria and cell lines. The oligonucleotide sequences, protein designations, and their relative molecular masses are presented in **Table S1** and in **Figure 1**, respectively. The proteins were expressed in the *Escherichia coli* BL21(DE3) strain, and were purified as detailed in the **File S1**. The expression of mCherry, OlyA-mCherry and mCherry-OlyA in the MDCK cell line was performed as described in **File S1**.

Protein concentrations were determined using a microvolume NanoDrop 2000 spectrophotometer (Thermo Scientific, USA). Protein size and purity were analysed using SDS-PAGE electrophoresis on homogenous 12% acrylamide gels. The proteins were stained with Coomassie blue. When transferred to polyvinylidene difluoride membranes (Millipore, Merck, USA) for Western blotting, the proteins were detected with mouse anti–penta-histidine antibodies (Quiagen, NL), anti-OlyA antibodies, or anti-phosphotyrosine antibodies (Sigma-Aldrich, USA), as appropriate. Other materials and analytical grade chemicals were from Sigma-Aldrich and Merck (USA).

### 2. Preparation of plasmids for the fluorescently labelled OlyA variants

An OlyA6 clone (henceforth, OlyA) from a cDNA library prepared from the total mRNA of *P. ostreatus* (strain Plo5 from the ZIM collection of the Biotechnical Faculty, University of Ljubljana, Slovenia) [39] was used to construct the recombinant OlyA variants as fusion proteins with the mCherry fluorescent protein, as described in **File S1**.

### 3. Preparation of large unilamellar vesicles

For the preparation of large unilamellar vesicles (LUVs) of various lipid compositions (cholesterol/SM [1∶1, mol∶mol], cholesterol/POPC [1∶1, mol∶mol], and POPC [100%]), lipid films were formed by removing the organic solvent from a lipid solution by rotary evaporation and vacuum drying. Lipids extracted from bovine erythrocytes [35] were dissolved in CHCl_3_∶MeOH (3∶1, v/v) at ∼10 mg/mL and vacuum dried. The lipids were swollen in 20 mM Tris-HCl, 140 mM NaCl, 1 mM EDTA, pH 8.0, at room temperature, to a final concentration of 10 mg/mL, and vortexed vigorously to give multilamellar vesicles. Large unilamellar vesicles of ∼100-nm diameter were prepared by exposing these multilamellar vesicles to eight cycles of freezing and thawing, and by extruding them through a 0.1-μm polycarbonate filter (Millipore, Merck, USA) mounted in a small-volume extruder (Avestin, Canada), as described previously [39], [40].

### 4. Haemolytic assay

Haemolytic activity was measured on a kinetic microplate reader (Dynex, USA) at ∼25°C, as described previously [41]. The density of the bovine erythrocyte suspension in erythrocyte buffer (140 mM NaCl, 20 mM Tris-HCl, pH 7.4) was adjusted to an apparent absorbance of 0.5 at 630 nm (A_630_). After the addition of serially diluted proteins in erythrocyte buffer, the decrease in apparent absorbance at A_630_ was measured for 30 min at 7 s intervals, to determine the t_50_; i.e., the time necessary for 50% haemolysis. The haemolytic activity was expressed as 1/t_50_ (min^−1^).

### 5. Surface plasmon resonance measurements

The kinetics of the interactions of the OlyA variants with LUVs composed of total erythrocyte lipids and with LUVs composed of mixtures of pure lipids (cholesterol/SM [1∶1, mol∶mol], cholesterol/POPC [1∶1, mol∶mol], or POPC [100%]) were monitored using a BiacoreX surface plasmon resonance (SPR)-based refractometer (Biacore AB, Uppsala, Sweden), and the data were processed with the BIA evaluation software (GE Healthcare, UK). The LUVs were captured on an L1 sensor chip (GE Healthcare, UK), and the experiments were run as described previously [42], [43] (for details, see **File S1**). Proteins (0.5 or 5 μM) were injected at a flow rate of 10 μL/min in Tris-HCl buffer (20 mM Tris-HCl, 140 mM NaCl, 1 mM EDTA, pH 8.0) as the running buffer. Sensorgrams were corrected for the untreated surface flow-cell response.

### 6. Cell culture

MDCK cells derived from a kidney of a normal cocker spaniel [44] (NBL-2, ATCC-CCL-34) were obtained from ATCC (Manassas, VA), and were maintained in a controlled atmosphere at 37°C and 5% CO_2_. The cells were grown on plastic dishes (TPP Techno Plastic Products, Trasadingen, Switzerland) and were subcultured using TrypLE Select (Gibco, Life Technologies, UK) when at 90% to 100% confluence. The control cell medium comprised A-DMEM/F12 (1∶1), 10% foetal calf serum, 50 U/ml crystacylin (Pliva, Croatia), and 50 U/mL streptofatol (Fatol, Griefswald, Germany). The transfection tool Lipofectamine LTX Reagent, culture media, and supplements were purchased from Invitrogen (Life Technologies, USA), unless otherwise stated.

### 7. Cell proliferation assay

Cell proliferation was measured using the colorimetric Cell Titer 96 AQueous One Solution Cell Proliferation Assay (Promega, USA) that uses 3-(4,5-dimethylthiazol-2-yl)-5-(3-carboxymethoxyphenyl)-2-(4-sulfophenyl)-2H-tetrazolium (MTS). This is based on the conversion of the tetrazolium salt into a coloured, water-soluble formazan product by the mitochondrial activity of viable cells at 37°C, where the amount of formazan produced by the dehydrogenase enzymes is proportional to the number of living cells. MDCK cells were plated in 96-well microtitre plates (Costar, USA) at a concentration of 1×10^4^ cells/well in growth medium (Gibco, Life Technologies, USA) with 10% foetal calf serum and antibiotics, at 37°C and 5% CO_2_. After 24 h the cells were treated for 1 h with native OlyA, OlyA-mCherry (C-terminal–tagged mCherry), or mCherry-OlyA (N-terminal–tagged mCherry) (all at 1 μM) dissolved in growth medium without foetal calf serum. Control cells were treated with growth medium only. One plate was then used for the MTS assay, while another plate was incubated for an additional 23 h. Before performing the MTS assay, the cells were washed three times with growth medium. For each well, 20 μL MTS reagent (CellTiter 96 AQueous Reagent, Promega, USA) was added directly to the cell culture. After 1 h, the absorbance was measured at 490 nm using an EL-800 microplate reader (BIOTEK Instruments, USA). The proliferation index was expressed as the ratio of the absorbance at 490 nm between the treated and the control cells (×100).

### 8. Binding of fluorescently tagged OlyA to MDCK cell membranes

The binding of fluorescently tagged OlyA to the membranes of MDCK cells was investigated using fluorescence microscopy. The cells were double labelled with OlyA-mCherry and membrane marker proteins (GST-lysenin, EqTII-Alexa488, D4-PFO-EGFP, CT-B-Alexa488, caveolin-1, flotillin-1). Additionally, internalisation of OlyA-mCherry into the cells was monitored. After growing the cells for 2 days on glass coverslips, fixed cells (4% paraformaldehyde) or living cells were incubated with OlyA-mCherry or with the negative control mCherry-OlyA (N-terminal–tagged mCherry), for different times at 37°C (**Tables S2** and **S3**). When the staining was performed on living cells, they were then washed with phosphate-buffered saline (PBS) and fixed in 4% paraformaldehyde (in PBS) for 20 min at 25°C. Double labelling with OlyA-mCherry and the lipid marker proteins (GST-lysenin, EqTII-Alexa488, D4-PFO-EGFP, CT-B-Alexa488) was performed by applying the mixture of OlyA-mCherry and the other protein in PBS to the fixed cells, with the double labelling with OlyA-mCherry and caveolin-1 or flotillin-1 performed on living cells (**Table S4**). To investigate the internalisation route of OlyA-mCherry, living cells were first exposed to 1 μM OlyA-mCherry for 10 min at 17°C (a temperature at which endocytotic processes are inhibited). Then, the cells were washed with PBS, supplemented with growth medium, and put into a cell incubator for different times, to follow endocytosis (as listed in **Tables S4 and S5**). After this, the cells were fixed with 4% paraformaldehyde for 20 min, washed with PBS, and then treated for 30 min at 37°C with the blocking/permeabilisation buffer (0.5% BSA, 0.02% sodium azide, 0.1% saponin, 0.1% gelatin and 50 mM NH_4_Cl, in PBS). The cells were then incubated with anti–caveolin-1, anti–flotillin-1, anti-clathrin, anti-EEA1, anti-cathepsin L, anti-GM130, anti-P230 or anti-giantin primary antibodies for 1 h at 37°C, as described in detail in **Table S5**. They were then washed with PBS and incubated with the appropriate Alexa Fluor 488-conjugated secondary antibodies (1∶500), for 90 min at 37°C. The coverslips were mounted in Vectashield with 4′,6-diamidino-2-phenylindole (DAPI) for nuclear staining, and analysed using an oil-immersion objective (63× oil/NA 1.40) under an AxioImager Z1 fluorescent microscope (Carl Zeiss, Germany) with an ApoTome device (Carl Zeiss, Germany) for the generation of optical sections. Images were acquired using the Axio-Vision programme (Carl Zeiss, Germany).

For the quantification of co-localisation, the Pearson's correlation coefficients were calculated from the raw optical sections for the pairing of OlyA-mCherry and the other markers. Background values were identical for all of the images. The Pearson's correlation coefficients were then calculated using the JaCoP plugin (Image J), from five images. The Pearson's coefficients were averaged, and a standard error of the mean was calculated. The degree of co-localisation from the Pearson's coefficients was categorised as described previously [45]: very strong (0.85 to 1.0), strong (0.49 to 0.84), moderate (0.2 to 0.48), weak/moderate (0.1 to 0.2), weak (−0.26 to 0.09) and very weak (−1 to −0.27)]. As we noted a number of the Pearson's coefficient estimates in the range between 0.1 and 0.19 that were significantly different from values >0.3, we arbitrarily introduced the additional category of “weak/moderate”.

We also investigated whether pre-treatment of living MDCK cells for 60 min with a cholesterol-scavenging agent, as 5 mM methyl-β-cyclodextrin (Sigma-Aldrich, USA), or for 30 min and 60 min with 0.5 U/mL sphingomyelinase from *Staphylococcus aureus* (Sigma-Aldrich USA) influenced the staining of these cells with OlyA-mCherry.

## Results

### 1. Production of recombinant proteins

The fluorescently labelled recombinant variants of OlyA and its thrombin-cleaved derivatives were initially prepared and characterised (see **Fig. 1** for structures, nomenclature, and Western blotting). The calculated *M*r of OlyA-mCherry-H_6_ and H_6_-mCherry-OlyA were 44,318.58 Da and 43,409.66 Da, respectively. According to the Western blotting, these proteins can be spontaneously cleaved (**Fig. 1B**) due to the instability of the mCherry protein at position 70, as previously reported [46]. This cleavage of the peptide bond, however, does not compromise the conformational stability and fluorescence characteristics of the fused proteins in solution.

We also inserted the *OlyA* gene into pmCherry plasmids, to be expressed as an mCherry fusion protein in the cytosol of the MDCK cells. The intracellular expression of the fluorescently fused OlyA variants was confirmed using Western blotting (**Fig. S1**).

### 2. Binding studies

**Figure 2:**
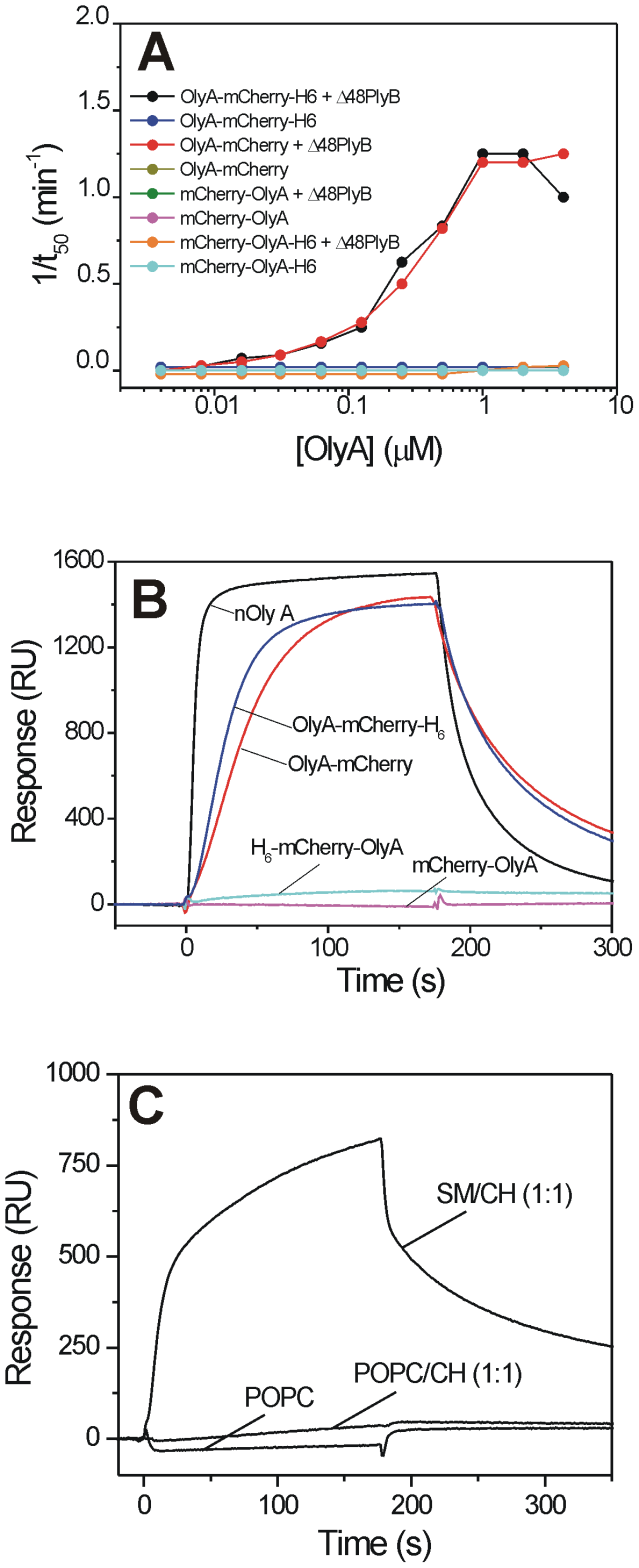
Membrane binding and permeabilisation by recombinant OlyA constructs. (**A**) Haemolytic activity of 16 nM Δ48PlyB supplemented with different concentrations of recombinant OlyA constructs, as indicated, expressed as the reciprocal of the half-time of haemolysis (1/t_50_). (**B, C**) Surface plasmon resonance analysis of interactions of OlyA variants (0.5 and 5 μM, respectivelly) with LUVs composed of total bovine erythrocyte lipids (**B**) and commercial lipid mixtures (**C**), in the molar ratios as indicated. For the surface plasmon resonance, the vesicles were immobilised on a Biacore L1 chip to 10,000±1,000 RU and the analytes were injected at a flow rate of 10 μL/min in running buffer (140 mM NaCl, 20 mM Tris-HCl, 1 mM EDTA, pH 8.0) at 25°C. Representative sensorgrams of triplicates are shown. SM, sphingomyelin; CH, cholesterol; POPC, 1-palmitoyl-2-oleoyl-*sn*-glycero-3-phosphocholine; nOlyA, native OlyA.

The functionality of the purified recombinant OlyA proteins was first investigated in terms of the promotion of haemolysis when combined with Δ48PlyB, and for the correct binding to lipid vesicles (**Fig. 2**). Notably, the use of N-terminal tags prevented the binding of these recombinant OlyA variants to lipids [27], and as such, together with Δ48PlyB, these proteins did not promote permeabilisation of lipid membranes at up to 4 μM (**Fig. 2A**). On the other hand, in combination with Δ48PlyB, the C-terminally tagged OlyA induced the typical sigmoidal time-course of haemolysis (data not shown), as reported before for the non-fused OlyA and Δ48PlyB [27]. Surface plasmon resonance studies of the interactions of these proteins (0.5 μM) using on-chip immobilised LUVs composed of total erythrocyte lipids provided additional data on their avidity for membrane lipids, as shown in **Fig. 2B**. The native OlyA and C-terminally tagged OlyA variants showed significant binding to the LUVs. The binding of these proteins to the erythrocyte lipid LUVs resulted in a maximal response, of 1400 RU to 1500 RU, while for the N-terminally tagged proteins the response was more than 10-fold lower. Further studies on the lipid specificity of OlyA-mCherry and its negative control, mCherry-OlyA (both at 5 μM) were performed by following their binding to LUVs composed of cholesterol/SM (1∶1, mol∶mol), cholesterol/POPC (1∶1, mol∶mol), or POPC (100%). The data clearly show interactions of OlyA-mCherry only with lipid vesicles composed of cholesterol and SM (**Fig. 2C**). The N-terminally tagged variant, mCherry-OlyA, did not bind to any of the tested LUVs (not shown).

### 3. Labelling of the MDCK cell plasma membrane with fluorescently tagged OlyA

The possibility to use the fluorescently labelled OlyA as a tool to study membrane domains with increased cholesterol/SM content in fixed and living cells was tested by examining the MDCK cells treated with either OlyA-mCherry or mCherry-OlyA (negative control) under fluorescence microscope.

#### 3.1. Staining of the fixed MDCK cells

**Figure 3:**
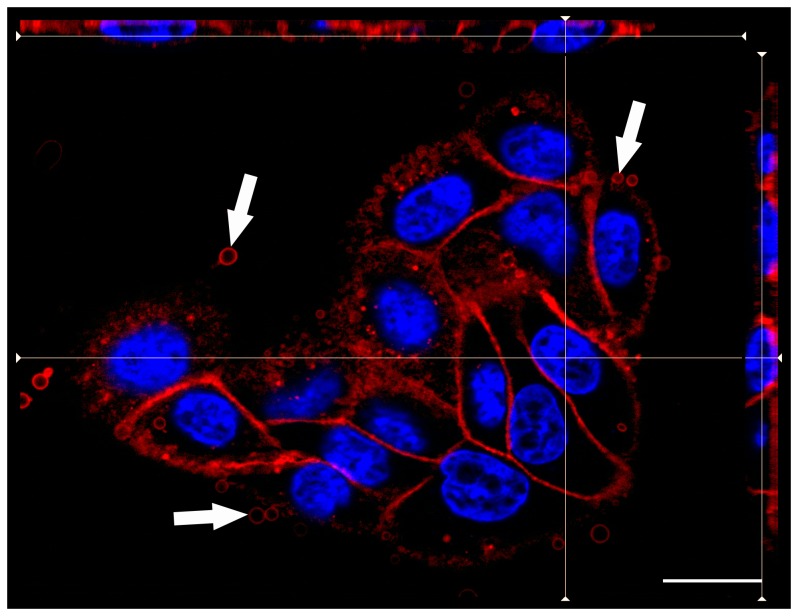
Labelling of fixed MDCK cells with OlyA-mCherry. Representative fluorescent image of fixed MDCK cells stained for 10 μM OlyA-mCherry (blue, DAPI; red, OlyA-mCherry). Vesicles appearing after OlyA-mCherry application are shown by arrows. Scale bar: 20 μm. Cells were stained as described in Materials and methods.

After growing the cells on glass coverslips for 2 days, they were fixed with 4% paraformaldehyde for 20 min, and then challenged with OlyA-mCherry (0.25–10 μM) or mCherry-OlyA (1 μM) for different times at 37°C, and washed with PBS (see **Table S2** for labelling conditions). In agreement with the haemolytic and SPR assays (**Fig. 2**), the fluorescent images showed that N-terminally tagged OlyA (mCherry-OlyA) does not bind to these fixed cells (data not shown). On the other hand, the OlyA-mCherry showed binding to the cell plasma membrane at all of the tested concentrations. The labelling of the plasmalemma of the fixed cells was uniform at 1 μM OlyA-mCherry (**Fig. 3**), and therefore this concentration was mostly used thereafter. However, using ≥1 μM OlyA-mCherry, there was formation of extracellular membrane vesicles, as indicated by the arrows in **Figure 3**.

#### 3.2. Staining of living cells

**Figure 4:**
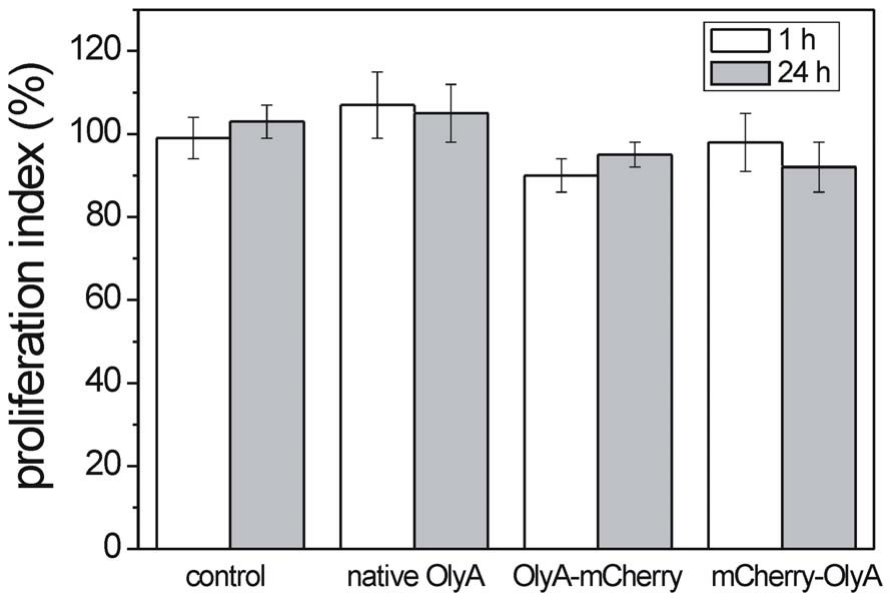
Effect of native OlyA and recombinant OlyA variants on survival of MDCK cells. Proliferation indices of MDCK cells treated with 1 μM proteins (as indicated), expressed as optical density of treated cells/optical density of control cells ×100%, after 1 h and 24 h exposure. Data are means ± SD from two independent experiments.

**Figure 5:**
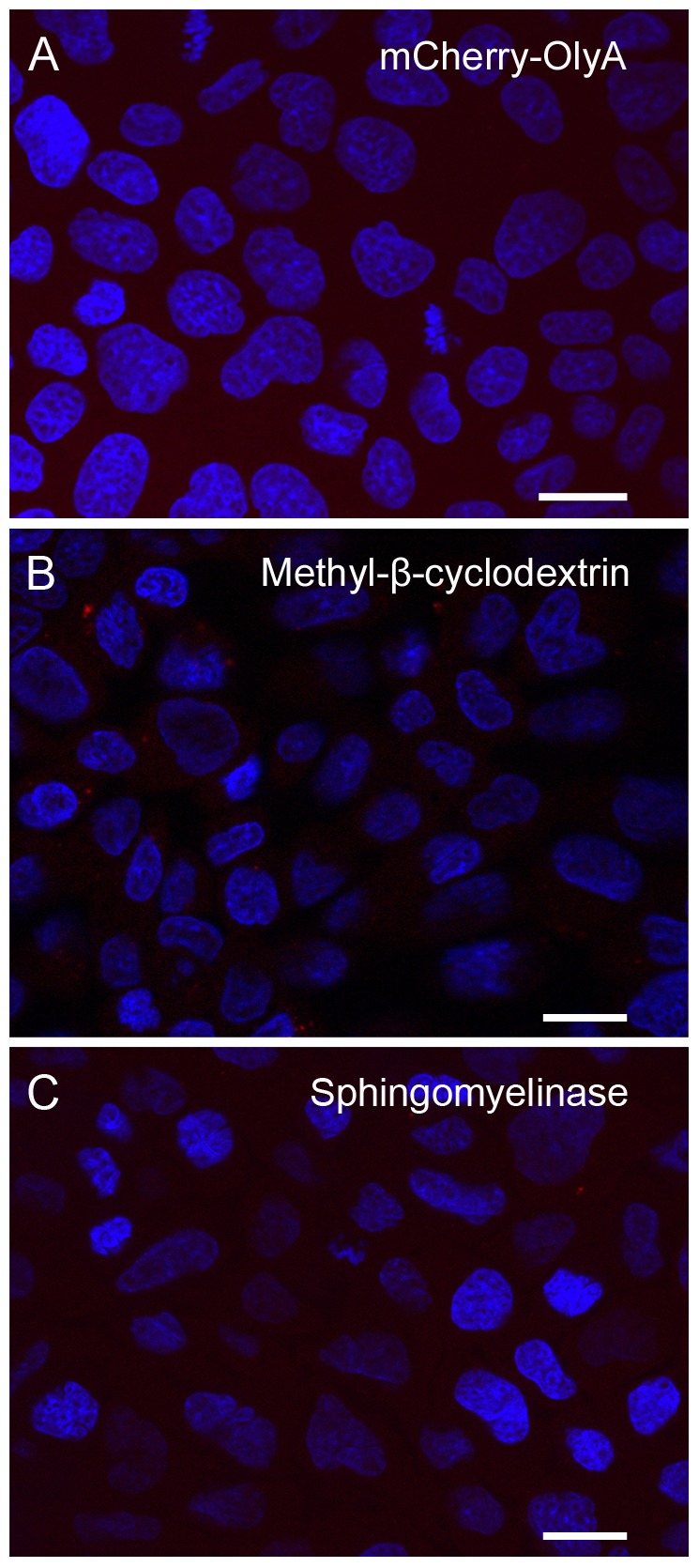
Binding of mCherry-OlyA, and effects of methyl-β-cyclodextrin and sphingomyelinase on binding of OlyA-mCherry to living MDCK cells. Representative fluorescent image of living MDCK cells grown at 37°C, showing no binding of 1 μM mCherry-OlyA after a 10-min incubation (A). No binding of 1 μM OlyA-mCherry was observed with cells pre-treated for 60 min with 5 mM methyl-β-cyclodextrin (B), or for 30 min with 0.5 U/mL sphingomyelinase (C). Scale bars: 20 μm. Cells were stained as described in Materials and methods.

**Figure 6:**
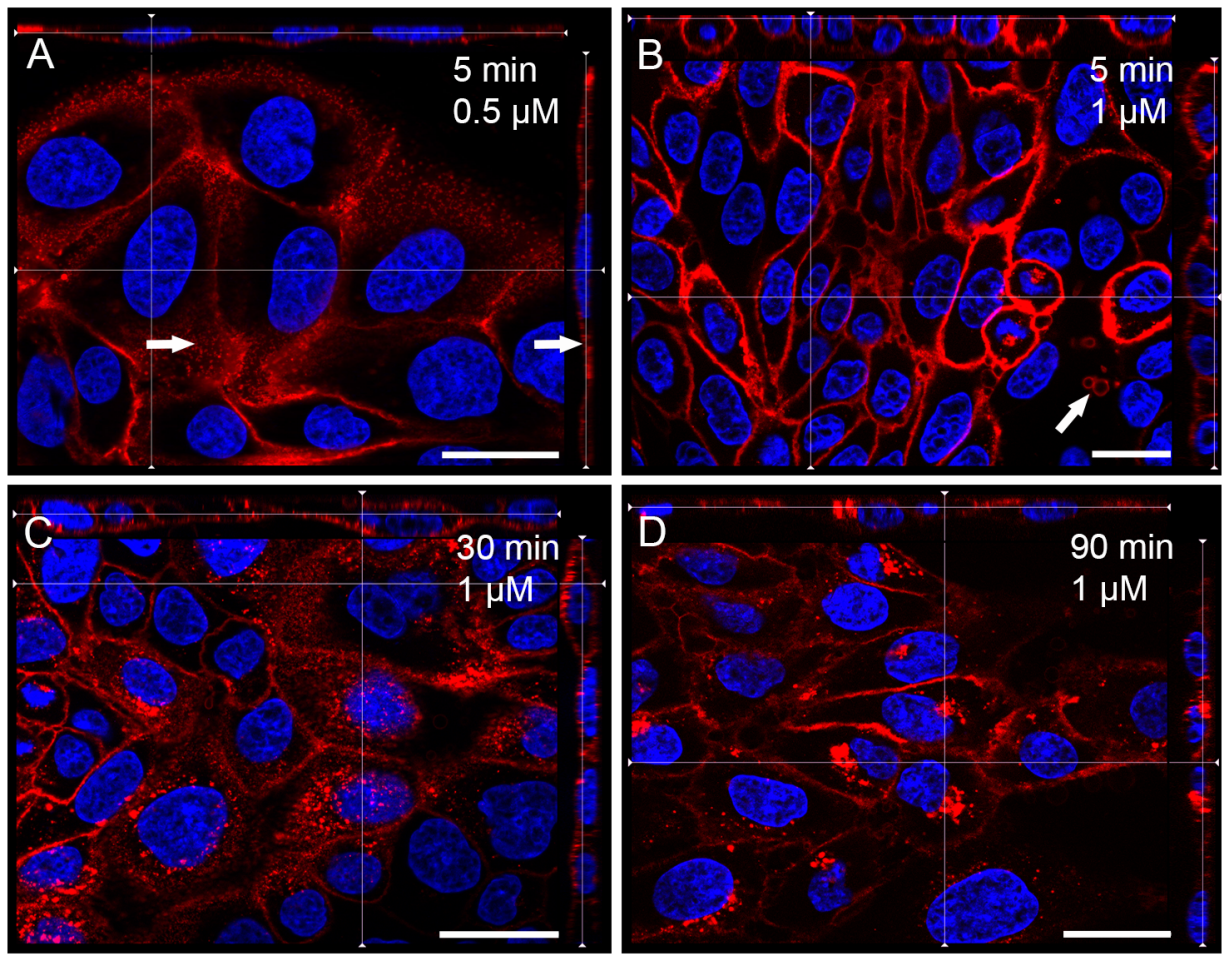
Labelling of living MDCK cells with OlyA-mCherry (blue, DAPI; red, OlyA-mCherry). Representative fluorescent images of living MDCK cells grown at 37°C after a 5-min incubation with 0.5 μM OlyA-mCherry (**A**), or after 10-min (**B**), 30-min (**C**), and 90-min (**D**) incubations with 1 μM OlyA-mCherry at 37°C. No budding of extracellular vesicles was detected on cells after 5 min of incubation with 0.5 μM OlyA-mCherry. Arrows, areas of flattened plasma membrane (**A**), or the individual extracellular vesicles after 5 min of 1 μM OlyA-mCherry application (**B**). Scale bars: 20 μm.

According to cell proliferation assays, at a final concentration of 1 μM, native (n)OlyA and fluorescently labelled OlyA variants did not induce any significant toxicity towards MDCK cells after 1 h and 24 h of incubation (**Fig. 4**). Non-confluent MDCK cells were grown on glass coverslips for 2 days and then treated with OlyA-mCherry (0.25–10 μM) or mCherry-OlyA (1 μM) for different times at 37°C (see **Table S3**). The cells were then washed with PBS and fixed with 4% paraformaldehyde. As in the case of the fixed cells (section 3.3.1), mCherry-OlyA did not bind to the plasma membranes of the living cells (**Fig. 5A**), with binding only observed with C-terminally tagged OlyA (OlyA-mCherry) (**Fig. 6A–D**). Pretreatment of the cells with methyl- β -cyclodextrin or with sphingomyelinase resulted in the total abolition of the OlyA-mCherry binding (**Fig. 5B, C**). Similar to the fixed cells, there was the formation of extracellular vesicles when the cells were treated with higher concentrations of OlyA-mCherry (≥1 μM). Nevertheless, based on microscopy observations (**Fig. 6D**), the cells remained alive after 90 min exposure to OlyA-mCherry, in agreement with the data from the cell proliferation assays (**Fig. 4**). The cells and their nuclei showed no morphological changes even after 3 days of incubation with 1 μM OlyA-mCherry (not shown). Furthermore, according to the phosphotyrosine assays, exposure of these cells to OlyA-mCherry does not induce inhibition or activation of cell-signalling pathways mediated by tyrosine kinases (**Fig. S2**). Fluorescent images of the cells grown at 37°C showed that 5 min incubation with 0.5 μM OlyA-mCherry was optimal for the labelling of the living cells, as OlyA-mCherry showed uniform binding to the plasma membrane, without the formation of extracellular vesicles (**Fig. 6A**). Incubation periods longer than 5 min or higher OlyA-mCherry concentrations led to the formation of extracellular vesicles, and several detached vesicles (2–10 μm diameter) were observed in the medium (**Fig. 6B**). Additionally, the OlyA-mCherry fluorescence was also detected inside these cells after an incubation of 30 min, as shown in **Fig. 6C**. After 90 min of incubation, OlyA-mCherry was concentrated near the cell nucleus (**Fig. 6D**). At this stage, there was only slight plasma membrane staining.

### 4. Distribution of OlyA-mCherry and other membrane marker proteins on MDCK cell membranes

**Figure 7:**
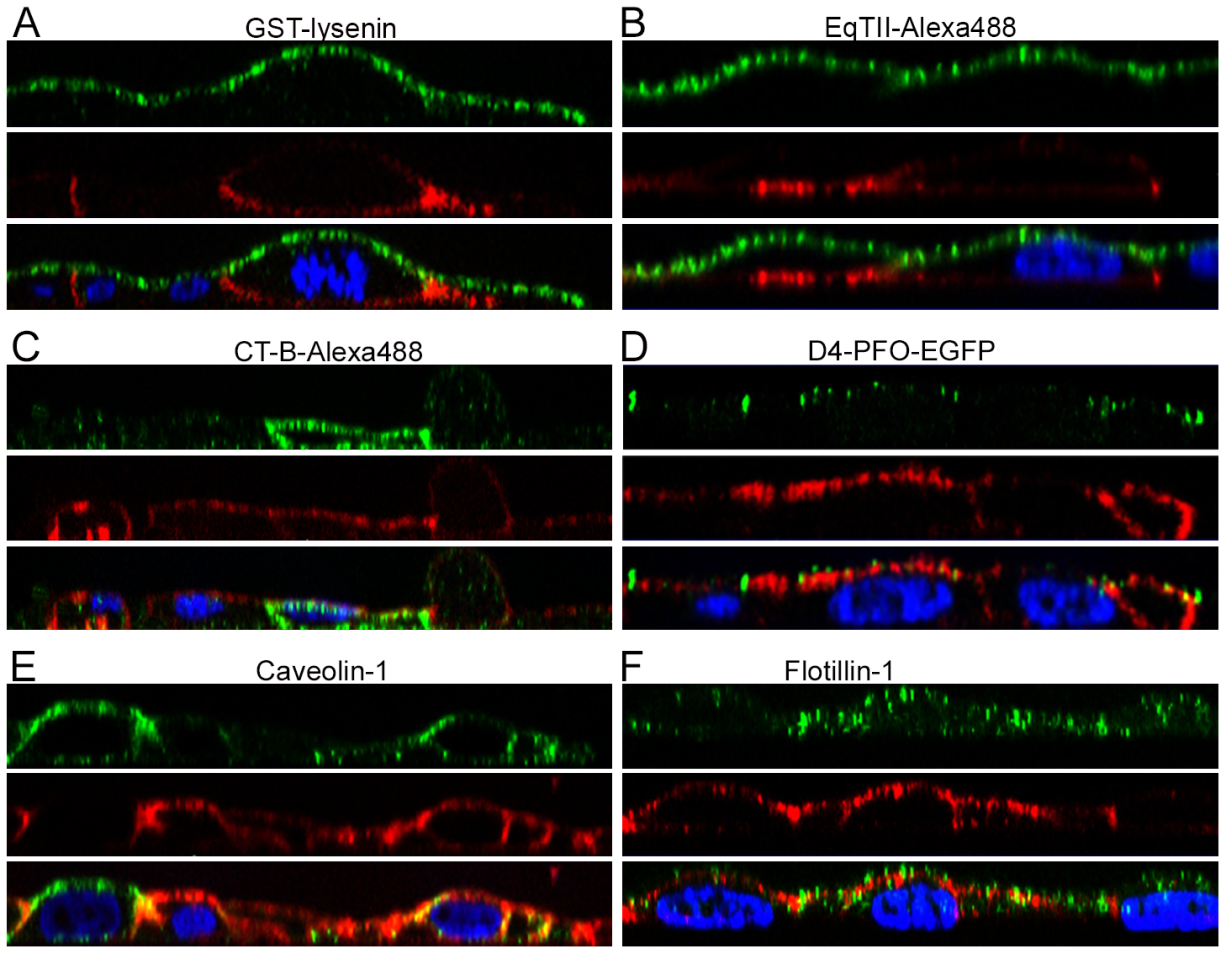
Double immunolabelling of MDCK cells treated simultaneously with OlyA-mCherry and toxin-derived or intrinsic proposed membrane markers (blue, DAPI; red, OlyA-mCherry). Representative fluorescent images from double labelling of raft markers (as indicated) (green) and OlyA-mCherry (red) was performed on fixed (A–D) and living (E, F) MDCK cells, as described in Materials and methods and Table S4. Cut views represent apical and basolateral distributions of OlyA-mCherry and the corresponding membrane markers. In all cases, the cells were exposed to OlyA-mCherry (1 μM) and to the other membrane markers for 10 min.

To investigate the binding pattern and distribution of OlyA-mCherry compared to other established toxin-derived lipid labels or intrinsic membrane raft markers, double fluorescent staining was performed on respectively fixed or living MDCK cells, as listed in **Table S4**. The levels of cell polarisation, and consequently the separation of the apical and basolateral plasma membranes, was monitored by immunolabelling of the tight-junction protein occludin (see **File S1**). Tight junctions were formed to a certain extent between the cells in confluent regions of the cell culture (**Fig. S3A**), although they were missing in cells with free lateral membranes (**Fig. S3B**). These bordering cells were consequently considered as non-polarised. OlyA-mCherry was distributed along the entire plasma membrane, and it did not show any clear co-distribution with toxin-derived lipid markers, in cells that were both living (not shown) and fixed (**Fig. 7A–D**). The GST-lysenin labelling was detected only at the apical plasma membrane in the polarised cells (**Fig. 7A**). The same labelling pattern was seen with another SM-sensing probe, Alexa-488 labelled EqTII (**Fig. 7B**). The ganglioside G_M1_- and cholesterol-binding probes, CT-B-Alexa-488 and D4-PFO-EGFP, respectively, gave very inhomogeneous labelling patterns. CT-B-Alexa488 had a similar distribution in these cells as OlyA-mCherry, as it was spread along the whole plasma membrane of the labelled cells (**Fig. 7C**). However, CT-B-Alexa488 labelled fewer cells when compared to OlyA-mCherry. The cholesterol-binding probe, D4-PFO-EGFP, predominantly labelled the apical cell membrane, with the labelling much weaker in basolateral membranes (**Fig. 7D**). Further immunodetection included two raft-associated transmembrane proteins involved in endocytosis: caveolin-1 and flotillin-1 (**Fig. 7E, F**). Double labelling revealed that OlyA-mCherry co-distributed particularly well with caveolin-1 at the plasma membrane, while its co-distribution with flotillin-1 was less evident.

**Figure 8:**
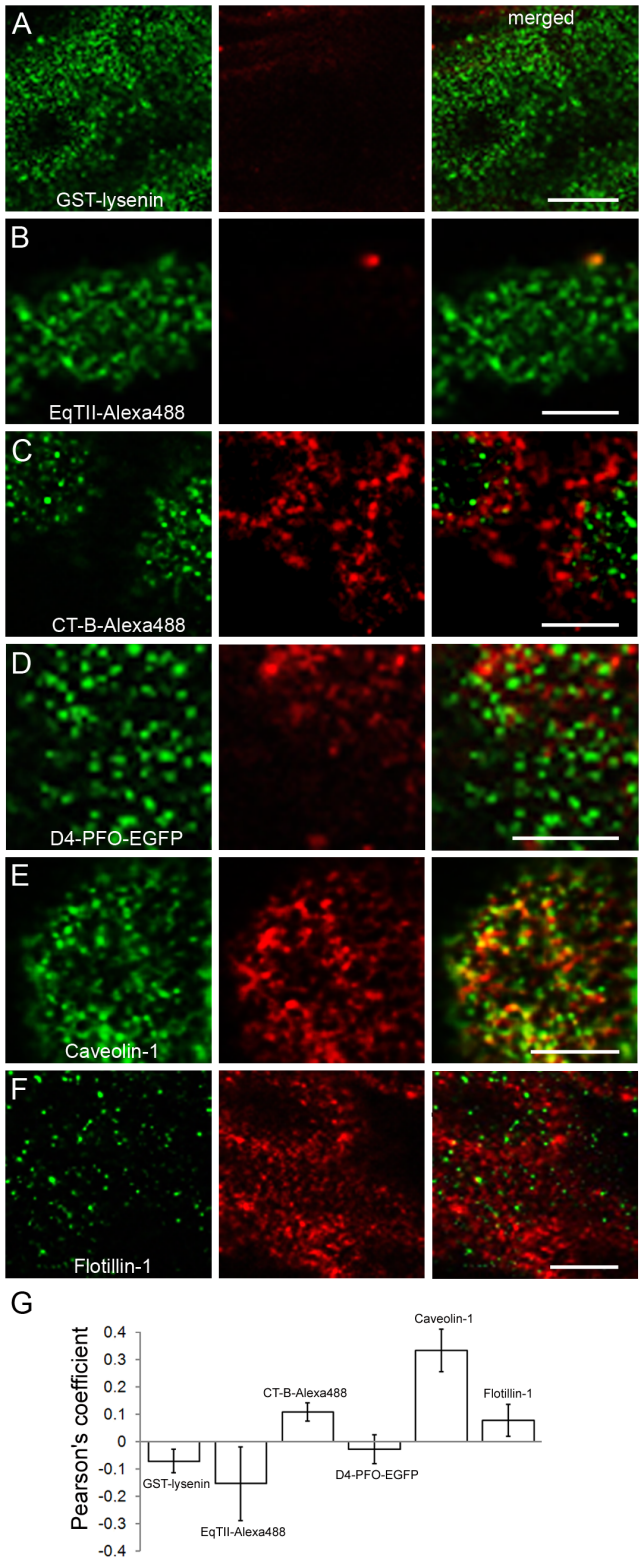
Co-localisation of OlyA-mCherry and other membrane markers. (**A–F**) Representative images of co-localisation between OlyA-mCherry (red) and other membrane markers (green), as indicated. Scale bar, 5 μm. (**G**) Quantitative analysis of the co-localisation between OlyA-mCherry and the other membrane markers. Pearson's correlation coefficients were calculated using the JACoP plugin (Image J programme) from optical sections. The degree of co-localisation from the Pearson's value correlation coefficients s were categorised as very strong (0.85 to 1.0), strong (0.49 to 0.84), moderate (0.2 to 0.48), weak/moderate (0.1 to 0.2), weak (−0.26 to 0.09), and very weak (−1 to −0.27). Error bars are the standard errors of the means (n = 5).

Further to these co-distribution studies (**Fig. 7**), the co-localisation was quantified (**Fig. 8**). To interpret the data of these co-localisation studies, the fuzzy linguistic system was used [45]. We compared the co-localisation of OlyA-mCherry and membrane marker labellings that were discernible as discrete spots at the apical plasma membrane. The membrane areas where co-localisation was measured were selected on the basis of the high intensity of labelling of markers that were correlated to OlyA-mCherry labelling. The highest degree of co-localisation was found between OlyA-mCherry and caveolin-1 (**Fig. 8E, I**), which was described with a quantifier of “moderate”. There was “weak/moderate” co-localisation between OlyA-mCherry and CT-B-Alexa488 (**Fig. 8C, I**), and no co-localisation of OlyA-mCherry with GST-lysenin, EqTII-Alexa488, or D4-PFO-EGFP (**Fig. 8A, B, D, I**), as also seen from the cut views of the cells (**Fig. 7**).

### 5. Internalisation of OlyA-mCherry in MDCK cells

**Figure 9:**
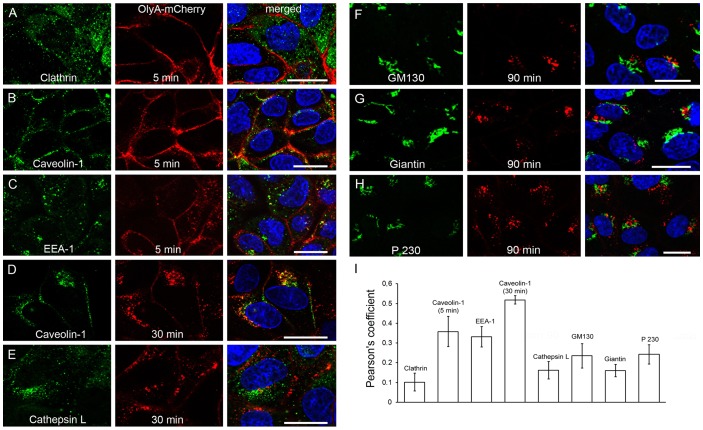
Internalisation of OlyA-mCherry in MDCK cells. (**A–H**) Representative fluorescent images of MDCK cells (blue, DAPI; red, OlyA-mCherry) showing immunolabelling of respective membrane domains (green, as indicated) and labelling of OlyA-mCherry (1 μM). After 5 min, OlyA-mCherry shows “weak/moderate” co-localisation with clathrin on the plasma membrane (**A**), and “moderate” with caveolin-1 (**B**) and EEA-1 (**C**). After 30 min, OlyA-mCherry shows “strong” co-localisation with caveolin-1 (**D**) and “weak/moderate” with cathepsin L (**E**). After 90 min, OlyA-mCherry shows “moderate” co-localisation with the GM130 (**F**) and P230 (**H**) markers of the Golgi apparatus, and “weak/moderate” for giantin (**G**). (**I**) Quantitative analysis of the co-localisation between OlyA-mCherry and the other membrane markers. Co-localisation in (F–H) are restricted to individual spots. Scale bars: 20 μm.

As the presence of OlyA-mCherry inside MDCK cells was observed at prolonged incubation times (**Fig. 6C, D**), we further investigated the pathways of OlyA-mCherry internalisation. To follow this, living cells were first pre-treated with OlyA-mCherry (see section 2.8 and **Table S5**), washed, incubated for different times to follow endocytosis, fixed with 4% paraformaldehyde, permeabilised, and labelled with appropriate antibodies. After 5 min of OlyA-mCherry internalisation, the cells were immunolabelled with anti-clathrin, anti-caveolin-1, and anti-EEA1 antibodies. At the plasma membrane, “weak/moderate” co-localisation was seen between OlyA-mCherry and the anti-clathrin antibody (**Fig. 9A, I**), while “moderate” co-localisation was seen for OlyA-mCherry with caveolin-1 (**Fig. 9B, I**) and EEA1 (**Fig. 9C, I**). The OlyA-mCherry signal was detected inside the cells after 30 min of internalisation (**Fig. 6C**), and therefore, we immunolabelled these cells with markers of the late endocytotic pathway: caveolar transport with an anti–caveolin-1 antibody, and late endosomes and lysosomes with an anti-cathepsin-L antibody (**Fig. 9D, E**). The highest co-localisation was seen for caveolin-1 and OlyA-mCherry, which after 30 min were concentrated together close to the nucleus (**Fig. 9D, I**). According to the Pearson's coefficients (>0.5), this co-localisation could be considered as strong. The cells that had internalised OlyA-mCherry for 90 min were further immunolabelled with markers of the Golgi apparatus, using antibodies against GM130 (marker of the *cis*-Golgi), giantin (marker of the *cis*- and medial Golgi, and involved in anterograde transport), and P230 (marker of the *trans*-Golgi network) (**Fig. 9F–H**). The co-localisation of the markers of the Golgi apparatus with OlyA-mCherry was “moderate” for GM130 and P230, and “weak/moderate” for giantin (**Fig. 9I**).

### 6. Expression of OlyA-mCherry in MDCK cells

mCherry, OlyA-mCherry and mCherry-OlyA were expressed in the MDCK cells to evaluate their binding to the cytoplasmic leaflets of the plasma membrane and to intracellular membranes. The proteins were seen to be expressed 4 h following transfection, although none of these proteins showed any clear membrane binding, even up to 4 days of incubation. All of these three proteins were uniformly distributed inside the cytoplasm (**Fig. S4**). Western blotting confirmed that the OlyA-mCherry expressed intracellularly remained intact for up to 2 days after its expression (data not shown).

## Discussion

To investigate the potential use of OlyA to visualise cellular membrane domains with increased cholesterol/SM content, which are typical of membrane rafts [3], [4], as a comparison with cholesterol-specific and SM-specific protein probes, we produced and characterised the 42.5-kDa fluorescent fusion protein, OlyA-mCherry. The closely identical aegerolysin PlyA2 was also recently fused with EGFP and reported to specifically bind cholesterol/SM-rich domains in membranes of HeLa cells [28].

The SPR and haemolytic assays revealed that this C-terminal tagging of OlyA with mCherry did not affect the lipid binding of the fusion protein. This is consistent with previous observations for both OlyA [27] and PlyA2 [28], where N-terminal tags obstruct the binding of these proteins to membranes. The OlyA-mCherry selectivity for the combination of membrane cholesterol and SM was very similar to that of unfused OlyA [27] and of PlyA2-EGFP [28], and this selectivity was confirmed by (i) showing its exclusive binding to equimolar cholesterol/SM LUVs in the SPR assay, and (ii) either depletion of the cell membrane cholesterol using methyl-β-cyclodextrin or enzymatic degradation of the cell membrane SM. Each of these treatments consistently abolished the OlyA-mCherry, and PlyA2-EGFP [28], binding to these membranes. C-terminal tryptophan residues, which are well conserved among PlyA, PlyA2, OlyA and EryA, might be responsible for the cholesterol/SM recognition and membrane binding of OlyA-mCherry, as was suggested by tryptophan-to-alanine point mutations of PlyA [28]. Furthermore, in these MDCK cells, OlyA-mCherry was not cytotoxic, as also confirmed for PlyA2-EGFP in HeLa cells and erythrocytes [28], and it did not stimulate or inhibit active signalling pathways that are mediated by tyrosine kinases. However, further studies are needed to fully explore any eventual effects of OlyA-mCherry, and of other aegerolysin-derived fluorescent protein chimeras, on other cell-signalling pathways.

At 37°C, OlyA-mCherry stained the membranes of fixed and living MDCK cells uniformly. This binding pattern is in contrast to previous studies using immunolabelled native sub-lytic OlyA/PlyB mixtures [47], [48], where selective binding and clustering of OlyA/PlyB was seen on the membranes of CHO and MDCK cells, and on mouse somatotrophs. The differences observed after using immunolabelled OlyA or its fluorescent OlyA-mCherry fusion derivative point to the caution that is needed in the interpretation of results obtained using different experimental approaches, as the immunolabelling, oligomerisation of antibodies, or formation of OlyA/PlyB oligomers can lead to different binding patterns. Similar uniform membrane binding of fixed HeLa cells was reported also for the related protein PlyA2-EGFP [28]; however, compared to the present study, PlyA2-EGFP was applied to the cells at approximately 50-fold lower concentrations. The differences observed in the detectability of these aegerolysin-derived cholesterol/SM fluorescent probes might derive from the different cell-membrane compositions of the cell lines tested. Indeed, cancer cells have been reported to have an increased content of cholesterol [49]–[51] and membrane raft domains [52], which might account for the observed differences.

Another interesting phenomenon observed after the exposure of fixed and living MDCK cells to the higher OlyA-mCherry concentrations was the formation of relatively large extracellular vesicles (2-10-μm diameter). This process, however, did not lead to cell death; indeed, we demonstrated that the cells remained living and metabolically active even after 24 h of exposition to 1 μM OlyA-mCherry. Formation of membrane blebs is a phenomenon that occurs in living cells during cell movement, under the influence of a number of factors, such as chemoattractants, and in the course of cytokinesis, cell spreading, and apoptosis. The formation of vesicles can also be induced by some viruses and bacterial cytolytic proteins [53]–[55]. The formation of these vesicles has been observed in cholesterol/SM 1∶1 vesicles exposed to recombinant OlyA [27]. Also, the present study shows vesiculation of living and fixed MDCK cells, and an unchanged morphology of these living cells after prolonged (up to 3 days) incubation with OlyA-mCherry. These observations suggest that this vesicle formation is a result of a direct physical effect of OlyA-mCherry on the plasma membrane, rather than of apoptosis. Similar observations and a similar explanation have already been reported for streptolysin O, a toxin that recognises membrane cholesterol and that can induce vesicle formation (ectocytosis) in living and formaldehyde-fixed mammalian cells [54]. In the study by Bhat et al. [28], there was no formation of membrane blebs upon application of PlyA-EGFP to HeLa cells, which was probably because of the application of substantially lower concentrations of PlyA-EGFP, as discussed above. Further studies to investigate the formation of membrane vesicles after cell treatments with the higher concentrations of OlyA-mCherry, and the composition of these vesicles, are currently in progress. However, as these vesicles are clearly stained with OlyA-mCherry, this suggests that they are enriched in cholesterol and SM.

To gain additional information about the OlyA-mCherry membrane binding, several double-labelling analyses were performed with these MDCK cells and OlyA-mCherry, combined with other molecules that are proposed to target specific raft-residing molecules. In the present study, we strictly distinguished between co-distribution and co-localisation of these membrane markers. Co-distribution was determined as the localisation of two markers at the cell level, which usually describes an equal or unequal distribution of the markers along the plasma membrane. However, it should be taken in consideration that the size of the individual rafts and the distances between neighbouring rafts are far below the spatial resolution limit of light microscopy [56], thus the co-localisation should not be misinterpreted as localisation of the two markers within a particular membrane nanodomain.

OlyA-mCherry showed different plasma membrane distributions compared to the cholesterol-binding probe PFO-D4-EGPF, and the SM-binding probes GST-lysenin and EqTII-Alexa488. Recent studies have indicated that the lysenin and EqTII derivatives do not even bind to the same population of SM in both the plasma membrane and intracellular membranes [23]. Furthermore, as in our earlier study where mouse somatotrophs were double immunolabelled with an OlyA/PlyB mixture and CT-B-Alexa488 [47], in the present study, OlyA-mCherry and CT-B-Alexa488 were seen to bind to different nanodomains of the MDCK cell membranes. In contrast to our double labelling with extrinsic raft-labelling probes, Bhat et al. [28] monitored the co-localisation of PlyA2-EGFP with intrinsic membrane molecules, and confirmed only partial co-localisation of PlyA2-EGFP with the raft-associated protein CD59 and the ganglioside GM3, and practically no co-localisation with the raft-excluded protein transferrin [28]. Altogether, this suggests that raft-like regions and other lipid nanodomains in the external leaflet of the plasma membrane are highly heterogeneous with regard to the local concentrations of cholesterol and SM, and their molar proportions, and it indicates the importance for the development of different cholesterol/SM probes for monitoring different sub-populations of lipid rafts. As reported for PlyA2-EGFP in HeLa cells [28], OlyA-mCherry expressed in these MDCK cells did not label the cytosol-exposed plasmalemma or intracellular membranes, which is consistent with the low contents of SM in cytosol-exposed membrane leaflets [57]. This is in contrast to the intracellularly expressed PFO D4 domain, fused with green fluorescent protein, which has been reported to label cholesterol-rich microdomains in the inner leaflet of the plasma membrane [58]. These differences confer both the lipid selectivity of the examined protein labels, and SM and cholesterol distribution in cellular membranes.

The investigation here of OlyA-mCherry co-localisation with intrinsic cell raft markers and its internalisation in MDCK cells after prolonged times of incubation, suggests that OlyA-mCherry is being associated and endocytosed into these cells *via* caveolin-1–mediated endocytosis, which is a raft-dependent pathway of internalisation. OlyA-mCherry highly co-localised with plasmalemmal caveolin-1, and not with flotillin, a non-caveolar raft marker, or clathrin [59]. This is consistent with the role of caveolae in the cellular recycling of cholesterol/sphingolipid-rich rafts [59] and the preference of OlyA-mCherry for combined cholesterol/SM. Within 90 min, the OlyA-mCherry–labelled membranes reached the juxtanuclear region of the Golgi apparatus. These data indicate the enrichment of cholesterol and SM in the intracellular membrane compartments, and they are in agreement with the very recent findings showing the enrichment of raft-associated cholesterol in endosomal fractions and in the *trans*-Golgi network [60]. In the study by Bhat et al. [28], where intracellular labelling of PlyA2-EGFP was studied for fixed and permeabilised HeLa cells, PlyA2-EGFP co-localisation was only seen with late endosome markers, and there was no co-localisation with early endosome and *cis*-Golgi markers. It appears that both of these fused proteins enter the cellular endosomal recycling system; however, further studies are needed to identify the sorting and characteristics of the vesicles labelled with these proteins. However, the transport of raft membranes labelled with OlyA-mCherry from the plasma membrane *via* caveole close to the Golgi apparatus is in agreement with the known formation of raft structures in the *trans*-Golgi using galectin, which is transported from the plasma membrane [61]. In contrast, another raft marker, CT-B, uses different raft-dependent and independent internalisation pathways to reach the endoplasmic reticulum and Golgi apparatus, which depend on the cell type [62], [63].

In conclusion, the fluorescent recombinant protein OlyA-mCherry shows potential as a marker for cholesterol/SM-rich membrane domains, such as rafts. OlyA-mCherry has a relatively small molecular weight, it is not cytotoxic, it has optimal fluorescence properties, and it is stable. OlyA-mCherry specifically and selectively senses the combination of the two most abundant and important raft-residing lipids, cholesterol and SM, and it does not oligomerise in the membrane. With some precautions noted, such as the use of lower concentrations to minimise membrane remodelling, OlyA and *P. eryngii* PlyA2 are suitable for further studies of membrane raft structure and function in fixed and living cells. They can also be used in diagnostics and studies of pathologies that are associated with disorders in lipid metabolism, such as Niemann-Pick syndromes A and B, or in the range of diseases that can arise from impaired cholesterol metabolism.

At the same time, our study clearly demonstrates that the fused cargo protein, i.e., mCherry, does not hamper OlyA membrane binding and its internalisation *via* caveolin-1–dependent endocytosis. Thus, another potential application for OlyA and *P. eryngii* PlyA2 deserves to be explored: their use as shuttles for delivering other fused proteins, such as enzymes, to early endosomes and to late caveolar compartments.

## Supporting Information

File S1 **Supporting experimental procedures.**
(DOCX)

Figure S1 **Western blotting of OlyA-mCherry, mCherry-OlyA and mCherry proteins expressed in MDCK cells.** Detection of proteins was performed using polyclonal anti-OlyA antibodies. Lane 1, MW markers; lane 2, OlyA-mCherry; lane 3, mCherry-OlyA; lane 4, mCherry.
(TIF)

Figure S2 **Effect of OlyA-mCherry on signalling pathways mediated by tyrosine kinases in MDCK cells.** MDCK cells were exposed to OlyA-mCherry (1 μM) for 10 min, and the detection of proteins in cell extracts was performed using Western blotting and antiphosphotyrosine antibodies. Lane 1, MW markers; lane 2, untreated cells; lane 3, cells treated with OlyA-mCherry.
(TIF)

Figure S3 **Immunolabelling of tight junctions in MDCK cells plated on glass coverslips for 2 days.** MDCK cells were plated on coverslips and grown for 2 days, and then immunolabelled with anti-occludin antibodies. Lines of occludin represent mature tight junctions as indication of cell polarization (**A**). Discrete spots of tight junction protein occludin (arrows) at the margins of cell culture represent tight junctions in the process of formation (**B**). The nuclei were labelled with DAPI. Scale bars: 20 μm.
(TIF)

Figure S4 **Intracellular expression of mCherry, OlyA-mCherry and mCherry-OlyA in MDCK cells.** Proteins coding for OlyA-mCherry, mCherry-OlyA and mCherry were expressed in MDCK cells, as described in the **File S1**. Scale bar: 20 μm.
(TIF)

Table S1 **Oligonucleotide primers used in this study.**
(DOCX)

Table S2 **Protocols for the labelling of fixed MDCK cells with OlyA-mCherry and mCherry-OlyA.**
(DOCX)

Table S3 **Protocols for the labelling of the living MDCK cells with OlyA-mCherry and mCherry-OlyA.**
(DOCX)

Table S4 **Protocols for double labelling of MDCK cells with OlyA-mCherry (1 μM) and the membrane marker proteins.**
(DOCX)

Table S5 **Protocols for OlyA-mCherry (1 μM) internalisation in MDCK cells.**
(DOCX)
