# Supplementary material for: Tracking Cholesterol/Sphingomyelin-Rich Membrane Domains with the Ostreolysin A-mCherry Protein
Source: PLoS One. 2014 Mar 24;9(3):e92783. doi: 10.1371/journal.pone.0092783 (PMC3963934; doi:10.1371/journal.pone.0092783)
Supplement: File S1 — Supporting experimental procedures. (DOCX) [file pone.0092783.s001.docx]

**SUPPORTING INFORMATION**

**Tracking cholesterol/ sphingomyelin-rich membrane domains with the ostreolysin A-mCherry protein**

**Matej Skočaj^1^, Nataša Resnik^2^, Maja Grundner^3^, Katja Ota^1^, Nejc Rojko^1^, Vesna Hodnik^1^, Gregor Anderluh^1,4^, Andrzej Sobota^5^, Peter Maček^1^, Peter Veranič^2^*, Kristina Sepčić^1^***

^1^ Department of Biology, Biotechnical Faculty, University of Ljubljana, Ljubljana, Slovenia

^2^ Institute of Cell Biology, Faculty of Medicine, University of Ljubljana, Ljubljana, Slovenia

^3^ Institute of Biophysics, Faculty of Medicine, University of Ljubljana, Ljubljana, Slovenia

^4^ National Institute of Chemistry, Ljubljana, Slovenia

^5^ Nencki Institute of Experimental Biology, Warsaw, Poland

**Methods**

1. Preparation of plasmids and proteins
   1. Preparation of plasmids for fluorescently labelled OlyA variants

The genes encoding mCherry linked to the C-terminus of OlyA (for OlyA-mCherry production) were cloned into the pET21c(+) bacterial expression vector using the *Nde*I, *Bam*HI and *Xho*I sites, and the genes coding for mCherry linked to the N-terminus of OlyA (for mCherry-OlyA production) into the pET8c(+) bacterial expression vector using the *Bam*HI, *Nde*I and *Mlu*I sites. This provided expression of the constructs without or with C-terminal or N-terminal hexahistidine (H_6_). Three plasmids were prepared to evaluate the binding of the OlyA-mCherry expressed to the cytoplasmic leaflets of intracellular membranes in the MDCK cell line. The gene coding for OlyA was cloned into the mammalian expression vectors pmCherry-N1 via the *Xho*I, *Bam*HI and *Sma*I sites and into pmCherryC1 via the *Bgl*II and *Bam*HI sites. The proteins mCherry, OlyA-mCherry and mCherry-OlyA were expressed in the MDCK cell line using Lipofectamine® LTX Reagent, according to the manufacturer instructions.

- 1. Expression, refolding and purification of fluorescently fused OlyA variants

The tagged OlyA variants were constucted as His_6_ (H_6_) fusion proteins, expressed in the BL21(DE3) *E. coli* strain that was transformed with the corresponding vector, and grown on LB plates with 100 µg/mL ampicillin (LBA). A single colony was inoculated into 100 mL LBA medium, and grown at 37 °C on a rotating wheel. The overnight culture (10 mL) was inoculated into 500 mL LBA medium. Typically, 3 L to 4 L of bacterial culture were grown at 37 °C up to an optical density (A_600_) of 0.6 to 0.8 at. After induction with 0.5 mM isopropyl β-D-1-thiogalactopyranoside, the bacterial growth was continued for the next 4 h to 5 h. The bacteria were centrifuged for 30 min at 4,370 *g* and 4 °C, and stored at -20 °C. The yield was 1.5 g to 2 g wet mass of cells/L. The bacterial pellet was kept on ice during homogenisation. The cells were resuspended at 0.5 g wet mass/mL in lysis buffer (50 mM NaH_2_PO_4_, 300 mM NaCl, 10 mM imidazole, pH 8.0), supplemented with lysozyme (0.5 mg/mL), DNAse (10 µg/mL), RNAse (20 µg/mL), benzamidine (1 mM), 4-benzenesulfonyl fluoride hydrochloride (0.5 mM), phenylmethylsulphonyl fluoride (0.5 mM), and β-mercaptoethanol (20 mM), shaken for 45 min at 4 °C, and sonicated (Vibracell, Sonics, USA) at 35% amplitude for 1 × 5 min on ice. The homogenate was centrifuged for 30 min at 26,323 *g* at 4°C. The supernatant was stored at 4 °C, and the pellet was washed with the same buffer (1 ml buffer/g dry weight bacteria). Shaking for 30 min on ice was followed by 3 × 1 min sonication and centrifugation under the same conditions. The supernatants were merged and filtered through a 0.2-µm cellulose-acetate filter and then loaded onto a 1.5-mL Ni^2+^-NTA column equilibrated in buffer A containing 50 mM NaH_2_PO_4_, 300 mM NaCl, 10 mM imidazole and 20 mM β-mercaptoethanol. Non-specifically bound protein was eluted with 50 mM NaH_2_PO_4_, 300 mM NaCl, 20 mM imidazole, pH 8.0, and the residual protein was eluted with 300 mM imidazole in the same buffer. When appropriate, the H_6_ tag was removed with 1 U/mg thrombin (Novagen, Merck, USA), at 22 °C for 16 h. Prior to this thrombin cleavage, the Ni^2+^-NTA elution buffer was exchanged with thrombin cleavage buffer (20 mM Tris-HCl, 150 mM NaCl, 2.5 mM CaCl_2_, pH 8.4) using Amicon ultra-4 (Ultracel-10k, Millipore, Merck, USA) centrifugal filters. After cleavage, the sample buffer was exchanged for 20 mM Tris-HCl, pH 8.0, and was used in the further purification on a MonoQ anion-exchange column (GE Healthcare, UK). The protein was eluted with a 0 mM to 150 mM NaCl gradient in the same buffer.

- 1. Expression, refolding and purification of EGFP-D4

The EGFP-tagged D4 domain of PFO was prepared by cloning the gene coding for D4 domain of PFO into the pET8c bacterial expression vector containing the EGFP gene, using the *Bam*HI and *Mlu*I sites. This provided expression of the construct without or with N-terminal hexahistidine (H_6_). All of the following procedures were identical to those described for the OlyA variants.

1. Surface plasmon resonance

Surface plasmon resonance was performed at 25 °C and a flow rate of 10 µL/min (except for deposition of LUVs, at 2 µL/min) in filtered and degassed 20 mM Tris-HCl, 140 mM NaCl, 1 mM EDTA, pH 8.0 as running buffer. The chip was equilibrated at room temperature for 30 min and cleaned with three 1-min injections of regeneration solution (100 mM NaOH:2-propanol, 2:3, v/v). The LUVs of various lipid compositions (1 mM lipid in running buffer) were injected across the sample and reference flow cell to reach approximately 10,000 ±1,000 RU. Loosely adsorbed lipids were washed out with two consecutive 1-min injections of 100 mM NaOH and the exposed lipophilic groups on the chip were saturated with a 1-min injection of 0.1 mg/mL bovine serum albumin in the running buffer, to minimize non-specific binding of protein to the microfluidic system.

1. Determination of the level of polarization of MDCK cells

MDCK cells were grown for 2 days on glass coverslips, washed with PBS, fixed in 4% paraformaldehyde (in PBS) for 20 min at 25 °C, and permeabilised with Triton X-100 (0.5%) for 5 min at 4 °C. The cells were the immunolabelled with primary rabbit anti-occludin (1:400) and secondary goat anti-rabbit Alexa 488-conjugated antibodies (1:500), to determine the tight junctions between the cells.

1. Anti-phosphotyrosine assay

Anti-phosphotyrosine assays [Cieśla et al., 2011] were carried out to investigate whether OlyA-mCherry binding influences signalling pathways in MDCK cells that are mediated by tyrosine kinases. The cells were grown on glass coverslips, and after 2 days they were exposed to 1 µM OlyA-mCherry for 10 min, detached, homogenised, and prepared for Western blotting.

Cieśla J, Frączyk T, Rode W (2011) Phosphorylation of basic amino acid residues in proteins: important but easily missed. Acta Biochim Pol 58:137-148.
