# Supplementary figures and images for: Tracking Cholesterol/Sphingomyelin-Rich Membrane Domains with the Ostreolysin A-mCherry Protein
Source: PLoS One. 2014 Mar 24;9(3):e92783. doi: 10.1371/journal.pone.0092783 (PMC3963934; doi:10.1371/journal.pone.0092783)

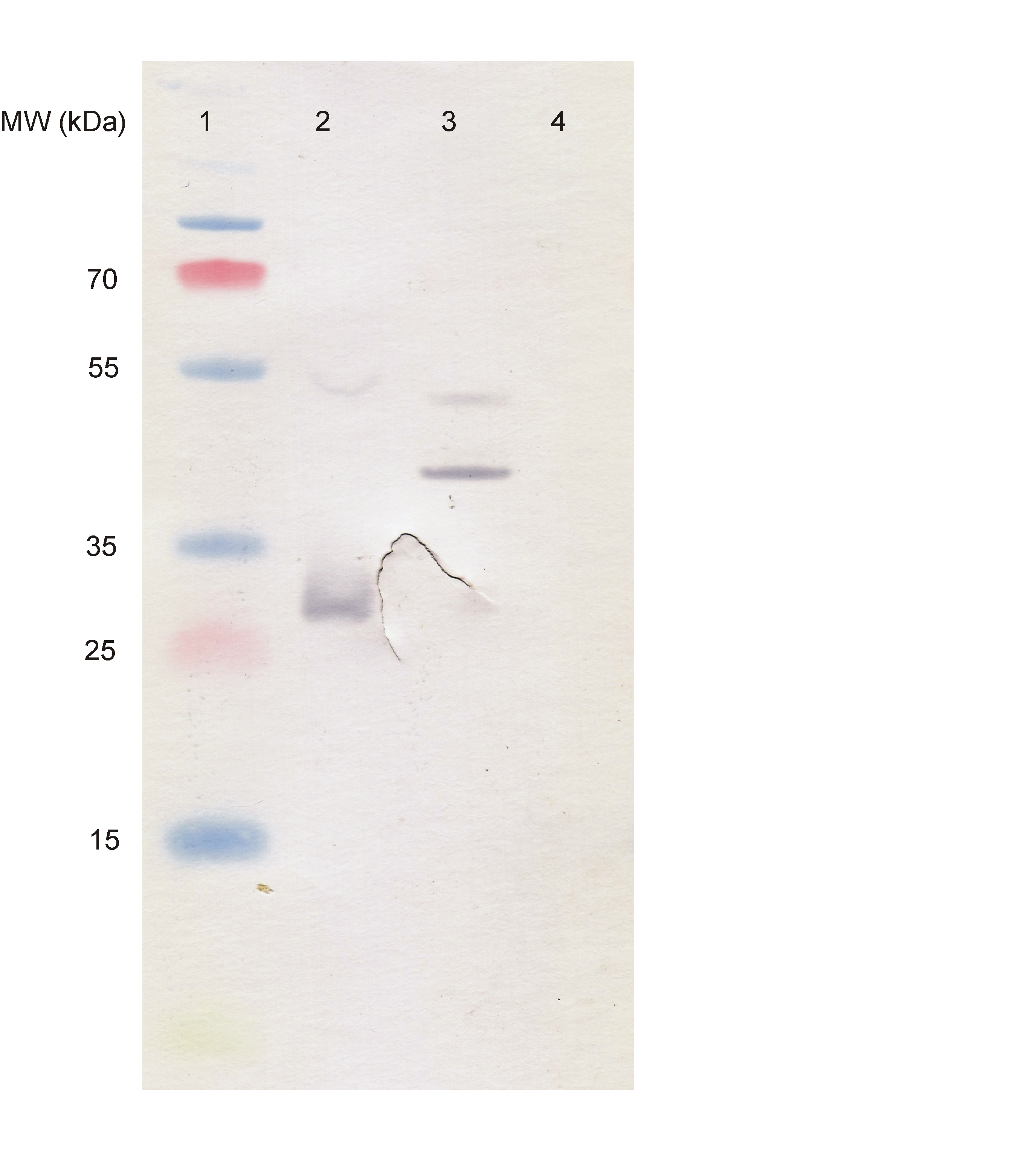

Supplement: Figure S1 — Western blotting of OlyA-mCherry, mCherry-OlyA and mCherry proteins expressed in MDCK cells. Detection of proteins was performed using polyclonal anti-OlyA antibodies. Lane 1, MW markers; lane 2, OlyA-mCherry; lane 3, mCherry-OlyA; lane 4, mCherry. (TIF) [file pone.0092783.s002.tif]

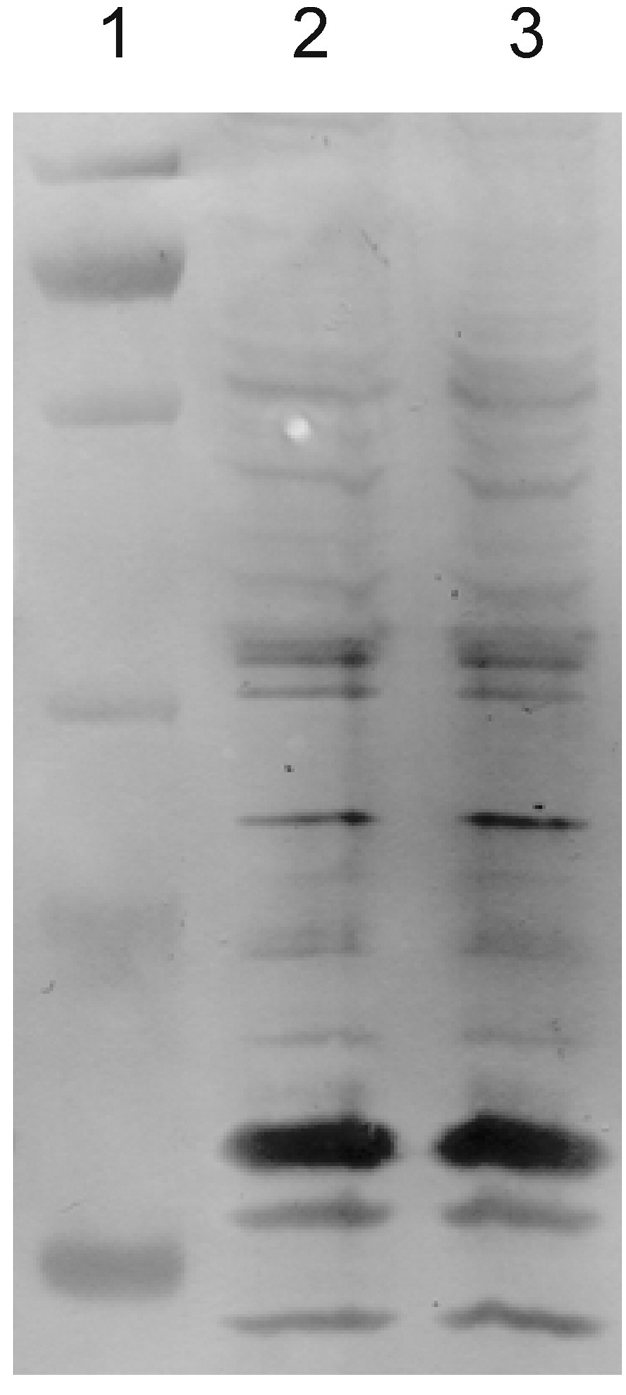

Supplement: Figure S2 — Effect of OlyA-mCherry on signalling pathways mediated by tyrosine kinases in MDCK cells. MDCK cells were exposed to OlyA-mCherry (1 μM) for 10 min, and the detection of proteins in cell extracts was performed using Western blotting and antiphosphotyrosine antibodies. Lane 1, MW markers; lane 2, untreated cells; lane 3, cells treated with OlyA-mCherry. (TIF) [file pone.0092783.s003.tif]

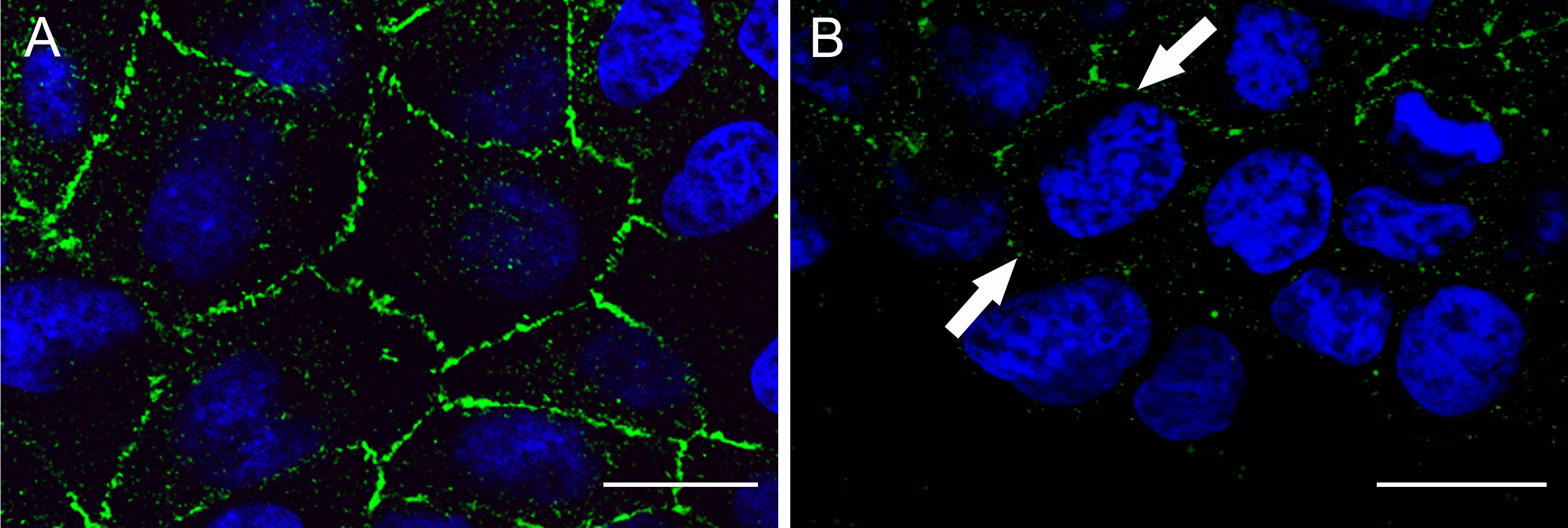

Supplement: Figure S3 — Immunolabelling of tight junctions in MDCK cells plated on glass coverslips for 2 days. MDCK cells were plated on coverslips and grown for 2 days, and then immunolabelled with anti-occludin antibodies. Lines of occludin represent mature tight junctions as indication of cell polarization (A). Discrete spots of tight junction protein occludin (arrows) at the margins of cell culture represent tight junctions in the process of formation (B). The nuclei were labelled with DAPI. Scale bars: 20 μm. (TIF) [file pone.0092783.s004.tif]

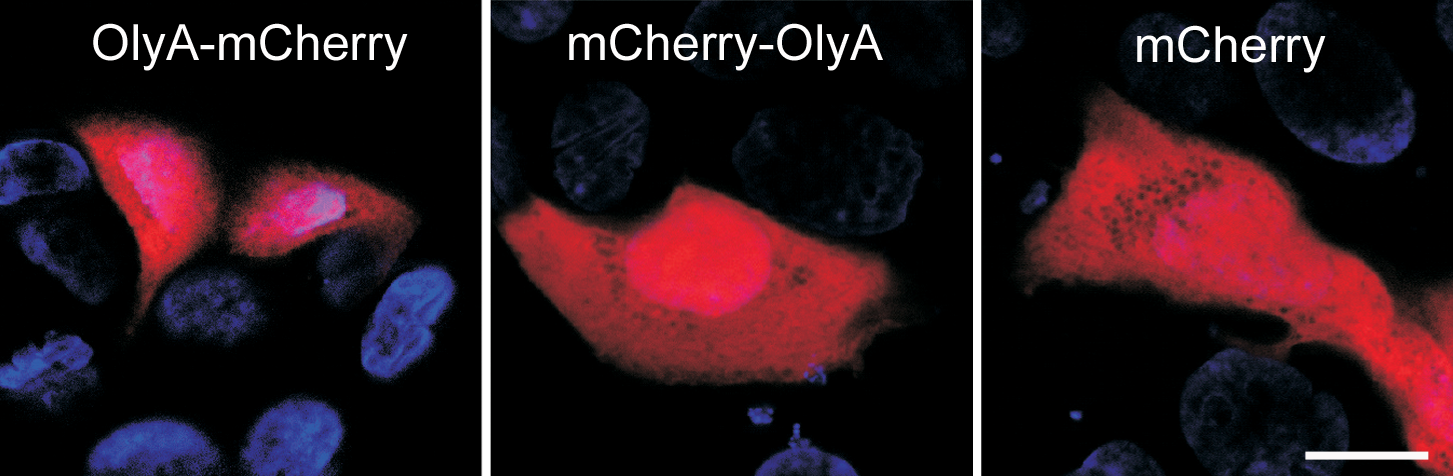

Supplement: Figure S4 — Intracellular expression of mCherry, OlyA-mCherry and mCherry-OlyA in MDCK cells. Proteins coding for OlyA-mCherry, mCherry-OlyA and mCherry were expressed in MDCK cells, as described in the File S1. Scale bar: 20 μm. (TIF) [file pone.0092783.s005.tif]
