# Supplementary material for: Tracking Cholesterol/Sphingomyelin-Rich Membrane Domains with the Ostreolysin A-mCherry Protein
Source: PLoS One. 2014 Mar 24;9(3):e92783. doi: 10.1371/journal.pone.0092783 (PMC3963934; doi:10.1371/journal.pone.0092783)
Supplement: Table S1 — Oligonucleotide primers used in this study. (DOCX) [file pone.0092783.s006.docx]

**Supporting Table S1**. Oligonucleotide primers used in this study.

| **Construct** | **5'-3' Forward primer** | **5'-3' Reverse primer** |
| --- | --- | --- |
| OlyA-mCherry-H_6_ | ttt ttt cat atg gcg tac gcc caa tgg gtc ATC | TTG aag aag ggg aac ggc agc gaa ggc aaa GGA TCC AAA AAA |
|  | aaa aaa gga tcc AGC agc ggc agc ggc GTG AGC AAG ggc gag gag | ATG GAC GAG CTG TAC AAG ctg gtt ccg cgc GGC AGC ctc gag ttt ttt |
| H_6_-mCherry-OlyA | aaa aaa Gga tcc GTG AGC AAG ggc gag gag | ATG GAC GAG CTG TAC AAG cat atg AAA AAA |
|  | aaa aaa cat atg gcg tac gcc caa tgg gtc | ACC TTG aag aag ggg aac TA ACG CGT TTT TTT |
| OlyA-mCherry | AAA AAA CTC GAG ATG gcg tac gcc caa tgg | acc ttg aag aag ggg aac T CCC GGG AAA AAA |
| mCherry-OlyA | TTT TTT AGA TCT gcg tac gcc caa tgg gtc | ACC ttg aag aag ggg aac TAG GGA TCC TTT TTT |

Abbreviations as in main text. Underlined: *Bam*HI, *Mlu*I, *Nde*I and *Xho*I restriction sites, respectively. Nucleotide sequences of all of the constructs were determined by MWG Operon (USA).
