# Supplementary material for: Tracking Cholesterol/Sphingomyelin-Rich Membrane Domains with the Ostreolysin A-mCherry Protein
Source: PLoS One. 2014 Mar 24;9(3):e92783. doi: 10.1371/journal.pone.0092783 (PMC3963934; doi:10.1371/journal.pone.0092783)
Supplement: Table S2 — Protocols for the labelling of fixed MDCK cells with OlyA-mCherry and mCherry-OlyA. (DOCX) [file pone.0092783.s007.docx]

**Supporting Table S2**. Protocols for the labelling of fixed MDCK cells with OlyA-mCherry and mCherry-OlyA.

| **Protein** | **Concentration**  **(**µ**M)** | **Incubation time**  **(min)** |
| --- | --- | --- |
| OlyA-mCherry | 0.25, 0.5, 1, 5, 10 | 10 |
| OlyA-mCherry | 1 | 10, 60 |
| mCherry-OlyA | 1 | 10 |
