# Supplementary material for: Tracking Cholesterol/Sphingomyelin-Rich Membrane Domains with the Ostreolysin A-mCherry Protein
Source: PLoS One. 2014 Mar 24;9(3):e92783. doi: 10.1371/journal.pone.0092783 (PMC3963934; doi:10.1371/journal.pone.0092783)
Supplement: Table S4 — Protocols for double labelling of MDCK cells with OlyA-mCherry (1 μM) and the membrane marker proteins. (DOCX) [file pone.0092783.s009.docx]

**Supporting Table S4**. Protocols for double labelling of MDCK cells with OlyA-mCherry (1 µM) and the membrane marker proteins.

| **Marker** | **Marker concentration** | **Incubation time**  **(min)** | **Cell labelling^a^** | **Primary and secondary antibodies (dilution)** |
| --- | --- | --- | --- | --- |
| CT-B-Alexa488 | 5 µg/mL | 10 | F | Rabbit anti–CT-B antibody (1:500);  Goat anti-rabbit Alexa488 antibody (1:500) |
| GST-lysenin | 1 µg/mL | 10 | F | Rabbit anti-GST antibody (1:500);  Goat anti-rabbit Alexa488 antibody (1:500) |
| EqTII-Alexa488 | 2.5 µg/mL | 10 | F |  |
| D4-PFO-EGFP | 2.5 µg/mL | 10 | F |  |
| Caveolin-1 | Intrinsic |  | L | Rabbit anti–Cav-1 antibody (1:200);  Goat anti-rabbit Alexa488 antibody (1:500) |
| Flotilin-1 | Intrinsic |  | L | Rabbit anti–Flot-1 antibody (1:200);  Goat anti-rabbit Alexa488 antibody (1:500) |

^a^, F, fixed cells labelled; L, live cells labelled
