# Supplementary material for: Tracking Cholesterol/Sphingomyelin-Rich Membrane Domains with the Ostreolysin A-mCherry Protein
Source: PLoS One. 2014 Mar 24;9(3):e92783. doi: 10.1371/journal.pone.0092783 (PMC3963934; doi:10.1371/journal.pone.0092783)
Supplement: Table S5 — Protocols for OlyA-mCherry (1 μM) internalisation in MDCK cells. (DOCX) [file pone.0092783.s010.docx]

**Supporting Table S5**. Protocols for OlyA-mCherry (1 µM) internalisation in MDCK cells.

| **Primary antibody target^a^ (1:200)** | **OlyA-mCherry preincubation (min)** | **Target system^b^** | **Secondary antibody (1:500)** |
| --- | --- | --- | --- |
| Clathrin heavy chain | 5 | E | Anti-mouse |
| Caveolin-1 | 5, 30 | E | Anti-rabbit |
| EEA-1 | 5, 30 | EA | Anti-rabbit |
| Cathepsin L | 30 | LE, L | Anti-mouse |
| GM130 | 90 | GA | Anti-mouse |
| Giantin | 90 | GA | Anti-mouse |
| P230 | 90 | GA | Anti-mouse |

^a^, antibody against specific intrinsic protein.

^b^, E, endocytosis; EA, early endosomes; LE, late endosomes; L, lysosomes; GA, Golgi apparatus.
